# Supplementary material for: Identification and Analysis of Sex-Biased MicroRNAs in Human Diseases
Source: Genes (Basel). 2023 Aug 25;14(9):1688. doi: 10.3390/genes14091688 (PMC10531062; doi:10.3390/genes14091688)
Supplement: Supplementary file 1 [file genes-14-01688-s001.zip › genes-2546597-supplementary.pdf]

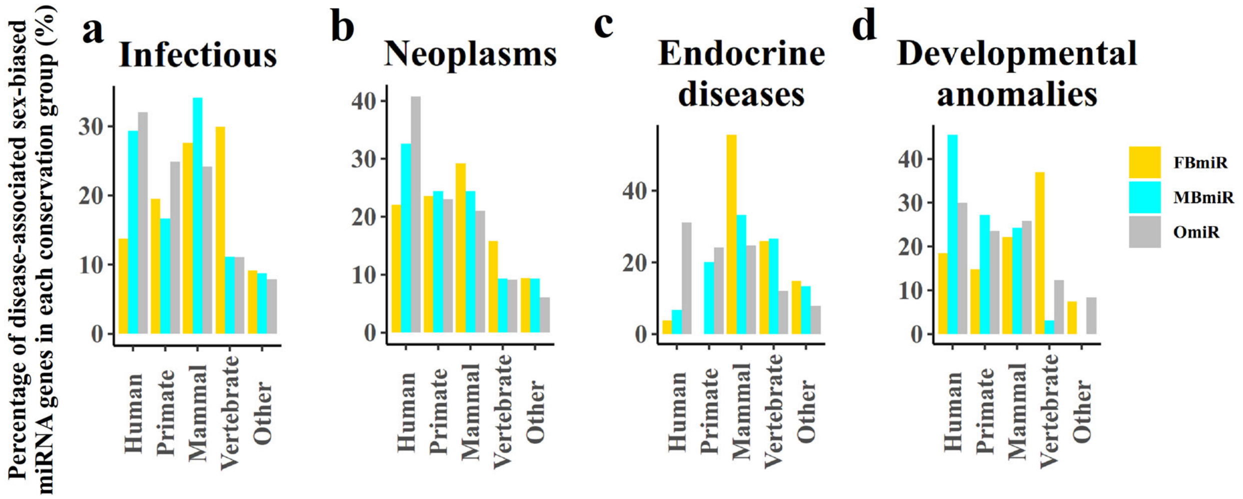

Figure S1. Percentage of disease-associated sex-biased miRNAs in each conservation group. Each bar in this chart represents the percentage of FBmiR or MBmiR in specific disease that fall in the corresponding conservation group for the four disease groups: **a.** Infectious **b.** Neoplasms **c.** Endocrine diseases, and **d.** Developmental anomalies.

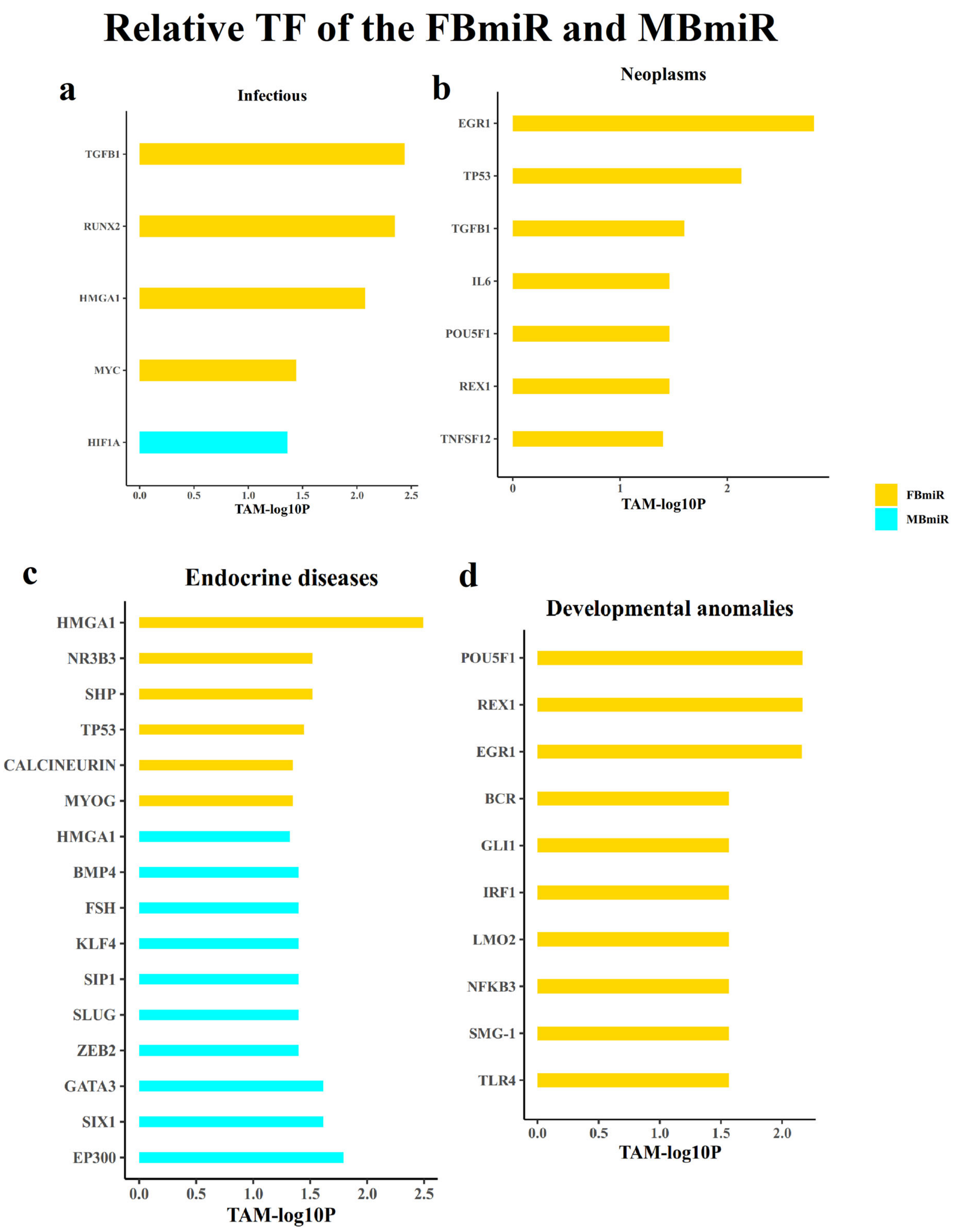

Figure S2. FBmiR- and MBmiR-related transcription factors (TF) enriched using the TAM2.0 algorithm in the four disease groups: **a**. Infectious **b**. Neoplasms **c**. Endocrine diseases, and **d**. Developmental anomalies.
